# Integrating genomics and genome editing for orphan crop improvement: a bridge between orphan crops and modern agriculture system

**DOI:** 10.1080/21645698.2022.2146952

**Published:** 2023-01-06

**Authors:** Huwaida Yaqoob, Arooj Tariq, Basharat Ahmad Bhat, Kaisar Ahmad Bhat, Iqra Bashir Nehvi, Ali Raza, Ivica Djalovic, PV Vara Prasad, Rakeeb Ahmad Mir

**Affiliations:** a Department of Biotechnology, School of Biosciences and Biotechnology, Baba Ghulam Shah Badshah University, Jammu and Kashmir, India; b Department of Bioresources, School of Biological Sciences, University of Kashmir, Srinagar, Jammu and Kashmir, India; c Department of Clinical Biochemistry, SKIMS, Srinagar, Jammu and Kashmir, India; d College of Agriculture, Fujian Agriculture and Forestry University (FAFU), Fuzhou, China; e Institute of Field and Vegetable Crops, National Institute of the Republic of Serbia, Novi Sad, Serbia; f Feed the Future Innovation Lab for Collaborative Research on Sustainable Intensification, Kansas State University, Manhattan, Kansas, USA; g Department of Biotechnology, School of Life Sciences, Central University of Kashmir, Jammu and Kashmir, India

**Keywords:** Climate change, CRISPR/Cas9, crop improvement, domestication, food security, future crops

## Abstract

Domestication of orphan crops could be explored by editing their genomes. Genome editing has a lot of promise for enhancing agricultural output, and there is a lot of interest in furthering breeding in orphan crops, which are sometimes plagued with unwanted traits that resemble wild cousins. Consequently, applying model crop knowledge to orphan crops allows for the rapid generation of targeted allelic diversity and innovative breeding germplasm. We explain how plant breeders could employ genome editing as a novel platform to accelerate the domestication of semi-domesticated or wild plants, resulting in a more diversified base for future food and fodder supplies. This review emphasizes both the practicality of the strategy and the need to invest in research that advances our understanding of plant genomes, genes, and cellular systems. Planting more of these abandoned orphan crops could help alleviate food scarcities in the challenge of future climate crises.

## GRAPHICAL ABSTRACT

**Figure:**
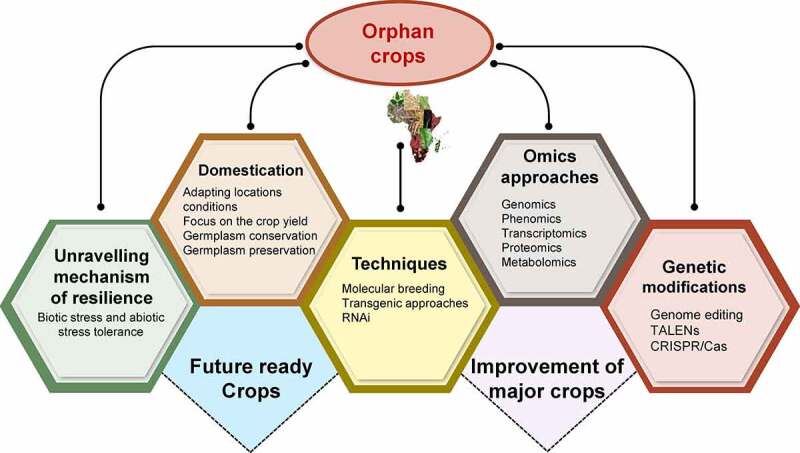

## 1. Introduction

Orphan crops are also known as ‘underutilized,’^1,^ ‘minor,’^2,^ ‘neglected,’^3,4^ ‘promising’ (for emerging markets or because of previously unrecognized valuable traits), ‘niche’ (of marginal importance in production systems and economies) and/or ‘traditional’ (used for centuries or even millennia) crops.^5^ These crops show many important characteristic features and are very much fitting to be grown as cash crops. Ironically, these crops are less familiar globally, primarily due to less attention paid by researchers, leading to inadequate or total lack of genetic and genomic resources. Despite the negligence in research and investment, they still have the potential to tackle multiple UN Sustainable Development Goals (such as zero hunger) in the low-income nations of Africa,^3^ Latin America, and Asia,^3^ and also in the growing western consumers interested in new healthier foods.^6^

Most orphan crops are resilient and can grow on extreme soil and climatic conditions as they possess relevant alleles and mechanisms to combat stress conditions,^7^ potentially lost from major crops.^8^ In due course of time, it has been recognized that orphan crops possess resilience traits^9^ and can be used to improve major crops as well as play an essential role in enhancing the sustainability of food systems^6,10,11^ which in turn has resulted in the launch of advanced research and development initiatives.^4^ It has also been seen that orphan crops possess other traits of importance that include nutrition,^6^ biofuel,^12^ medicinal value,^13^ cosmetics,^14^ and feed/fodder.^15^

**Figure 1.**
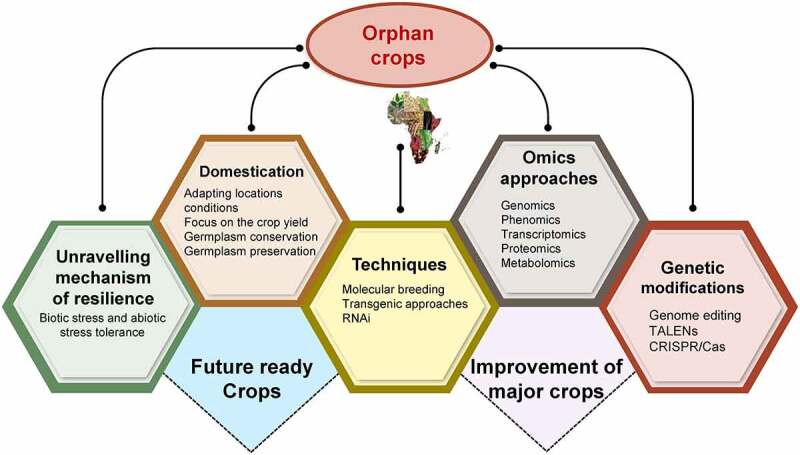
General considerations and future strategies employed for development of resilient crops using wild repository of orphan/underutilized crops.

The research and breeding efforts were majorly focused on some of the few crops on which the world’s food supply relies, like rice, wheat, maize, soy, and potato. For instance, by identifying the wild rice allotetraploids, the important wild sources of *de novo* domestication can be used by tissue culture, transfronation and genome editing systems to domesticate the wild rice races.^16^ In addition to these crops variety of orphan crops was also grown by small farmers.^17,18^ Orphan crops are a set of nutritious, tasty, and well-adapted species but, due to wild characteristics, are often unsuited to intensive agriculture.^19,20^ Orphan crops possess many characteristics and can be a lifesaver to the millions who go to bed hungry every night.^20^ Besides, orphan crops served as resource material for agricultural research to further increase the stress tolerance of major food crops and also brought diversity to agriculture.^20^ In addition, it also favored shifting food habits of modern agriculture. It has been proven that most orphan crops such as buckwheat (*Fagopyrum esculentum*), cassava, banana, and quinoa are not only important for food and feed but also hold great potential for industrial applications, effective management and have contributed toward poverty reduction and global food security.^21,22^ Conventional breeding for quality enhancement in orphan crops is challenging, while genetic manipulation via guided nucleases has proven an ideal platform for improving orphan crops^23–25^ (Fig. 1). This robust domestication was used to tackle changing climate scenarios and growing food security issues. Therefore, the current review aims to emphasize the practicality of the strategy and the need to invest in exploring the genomes, proteomics, and other omics studies to unravel their potential as frontline crops. In our investigation and exploration, we have discussed the updated account on orphan crops and their potential to act as buffering agents for securing nutrition and food security at global level. More importantly, we have detailed their social, ethical and legal point of consideration to have a global overview to make sure their role as multidimensional crops for future.

## 2. Orphan Crops: A Rich Repository Crop for Food and Nutritional Security

Orphan crops (crops for the future) are cultivated in very limited regions at global level. The reason for this low cultivation may be due to less research involved in to unravell their resilient characteristics.^4,6,26,27^ The worldwide economic value of orphan crops is limited but is extremely important at local levels, especially in developing countries.^9^ These crops mainly include cereals, pseudo cereals, legumes, and root crops, contributing to food and nutritional needs worldwide. These crops possess many properties like being resilient to biotic and abiotic stresses, high nutritional and medicinal value, and high photosynthetic efficiency. Examples of orphan cereals that utilize highly efficient C4 photosynthesis pathways include foxtail millet, pearl millet, broomcorn millet, barnyard millet, fonio millet, adlay, finger millet, and tef.^28–30^ C4 photosynthesis promotes the efficient use of nitrogen and water by reducing photorespiration to a minimum in hot and arid climates compared to C3 photosynthesis. Wild relatives also adopt the C4 pathway.^31^ Some of the orphan crops and their characteristic properties are given in Table 1.

**Table 1.** Showing the detail of major orphan crops and their characteristic traits considered for domestication process.

Orphan crop	Scientific name	Characteristic trait	References
African rice	*Oryza glaberrima*	Stress tolerance	^32^
Amaranth	*Amaranthus* spp.	Nutrition	^33^
Bambara groundnut	*Vigna subterranean*	Nutrition; Drought tolerance	^34^
Barnyard millet	*Echinochloa crusgalli*	Abiotic stress tolerance	^35^
Buckwheat	*F. esculentum*	Nutrition	^36^
Cassava	*Manihot esculentum*	Drought tolerance	^37^
Chickpea	*C. arietinum*	Nutrition	^38^
Cowpea	*Vigna unguiculata*	Nutrition; Drought tolerance	^39^
Enset	*Ensete ventricosum*	Drought tolerance	^40^
Foxtail millet	*Setaria italica*	Abiotic stress tolerance	^41^
Grass pea	*Lathyrus sativus*	Nutrition; Extreme drought tolerance	^42^
Horsegram	*Macrotyloma uniforum*	Nutrition	^43^
Kodo millet	*Paspalum scrobiculatum*	Abiotic stress tolerance	^43^
Lentil	*Lens culinaris*	Nutrition	^44^
Linseed	*Linum usitatissimum*	Nutrition	^45^
Little millet	*Panicum sumatrense*	Abiotic stress tolerance	^35^
Okra	*Abelmoschus esculentus*	Nutrition; Biotic stress tolerance	^46^
Pearl millet	*Pennisetums glaucum*	Abiotic stress tolerance	^47^
Pigeon pea	*Cajanus cajan*	Nutrition	^48^
Proso-millet	*Panicum miliaceum*	Abiotic stress tolerance	^41^
Quinoa	*C. quinoa*	Nutrition	^49^
Sweet potato	*Ipomoea batatas*	Nutrition	^50^
Tef	*Eragrostis tef*	Gluten-free; Abiotic stress tolerance	^51^
Yam	*Dioscorea* spp.	Drought tolerance	^52^

Orphan crops, as already discussed, possess many important features and can be a lifesaver to the millions of hungry humans on global scale. As stress-tolerant in nature, these crops can grow under unpredictable changing climates and ensure yield when major crop varieties fail, thus ensuring food security for small farmers. Besides, orphan crops served as resource material for agricultural research to further increase the stress tolerance of major food crops and also brought diversity to agriculture. In addition, these crops also favored shifting food habits of modern agriculture.^31,53^ It has been seen that orphan crops and their wild relatives possess many beneficial traits compared to major crops, which can be used to overcome different problems of modern agriculture and help make the practice more sustainable. Since most of these crops were well suited to grow in poor and marginal lands, there was little need for irrigation and fertilizers to boost their productivity. Coupling these crops with various sustainable agricultural techniques such as crop rotation and intercropping have been proven to be the best approaches to maintain a high yield of industrial agriculture while having a minimal ecological impact.^54^ Intercropping of some orphan crops like quinoa with beans, corn, and tarwi improved its yield and helped in pest control.^55^ Crop rotation of broomcorn millet with other crops prevented diseases/posts, helped in weed control, and helped maintain soil moisture.^56^

**Figure 2.**
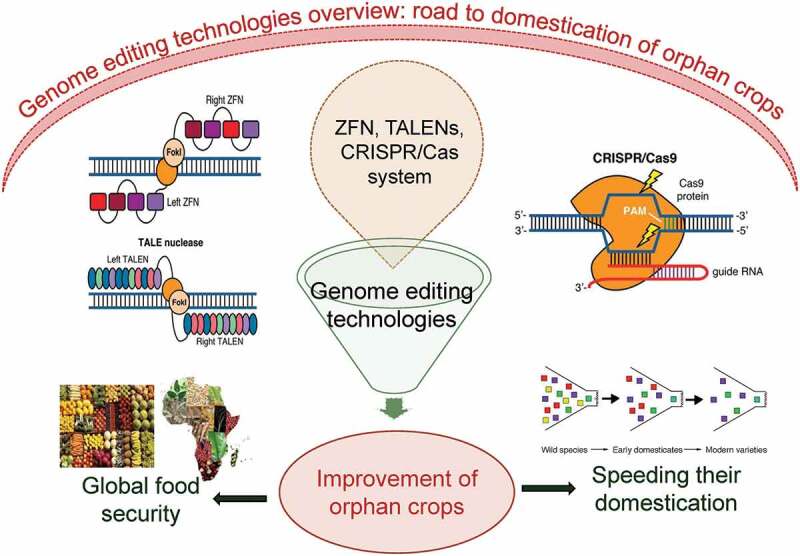
The figure shows the use of genome editing technologies applied for speeding up the domestication process such that food security will be attained in future.

Despite possessing these properties, some orphan crops are still not domesticated and possess a lot of potential in terms of quality and quantity of yield. Domestication of various crops through past approaches can lead to the faster improvement of orphan crops.^57^ In addition to these approaches latest developing techniques like high-throughput sequencing and genome editing can play an important role in the genetic improvement and manipulation of these minor crops. These crops possess many properties like being resilient to biotic and abiotic stresses, high nutritional and medicinal value, and high photosynthetic efficiency. As orphan crops are already comparatively more stress tolerant and nutritious, domestication efforts must mainly concentrate on boosting the yield.^49,57^ Some gene-controlling characteristics often conserved include grain size, weight, and height, and they can be directly targeted using advanced genome editing techniques like clustered regularly interspaced short palindromic repeats (CRISPR/Cas9) systems (Fig. 2). Sequencing the genome of these crops leads to large-scale improvement of these crops and makes them available publicly. In addition, this also served as a solid foundation for the modifications and development of these crops via various techniques like molecular breeding and genome editing.^49^

## 3. Genomic Information on Orphan Crops

The molecular breeding has been revolutionized due to the advance in genomics and genome editing technologies.^58–61^ The valuable information of genomic data gained from high-throughput sequencing and computational analysis can be accomplished to identify desirable traits incorporated in wild relatives of crop plants.^59,61^ Many initiatives have been taken for advanced research and development of orphan crops. Different institutions have taken the step for the detailed analysis of some crops; 101 orphan crops have been selected by African orphan Crops Consortium (AOCC) for detailed studies and whole-genome sequencing.^62^ Next-generation sequencing, in which whole-genome sequencing is also available for many crops and their wild relatives, has proven an efficient way of facilitating the domestication of genes. Thus, crop improvement has been “democratized.”^63^ The availability of whole-genome sequencing provides information about ortholog identities and selective sweeps and is an essential requirement for both source and recipients of allelic variance. In addition, this application of high-quality sequencing provides necessary information about three features of domestication, which are genomic structural variation, transposable elements, and gene as well as whole-genome duplications.^64^ Studies have shown that genomic structural variation concerns some important agricultural traits, copy number variation like variation in vernalization and flowering time in wheat^65^ and barley freezing tolerance.^66^ At high frequencies, transposable elements are present all over the whole genome.^67^ Whole-genome duplication has also proven significant by revealing almost 200 crops and over 2000 wild species.^68^ In recent years, significant achievements in genome sequencing have been achieved in the grass family, and reference genomes of at least 11 cereals have been published. The orphan cereal which was first sequenced was foxtail millet.^69^ Sequencing of orphan legumes has also been done, and at least the genome of 16 orphan legumes have been sequenced; the orphan legume whose genome was first sequenced was pigeon pea.^26^ The genome of other orphan crops, which has also been sequenced, includes pseudocereals amaranth,^70^ buckwheat, and quinoa^71^ and the root crops cassava,^72^ sweet potato,^73^ and yams.^74^ Besides genome sequencing, transcript profiling, epigenetics studies, and the use of metabolomics also provide considerable insight into domestication.^57,64,75^

## 4. Omics Studies and Their Utilization for Desired Genome Editing

Genome sequencing technologies have undergone rapid evolution, which enables the explosion of large-scale data at each level of information that is from gene sequence, transcriptome, proteome, epigenome as well as metabolite patterns that usually determine the variability in cellular networks and functions at the systems level.^75–79^ The information gained by the multi-omics approach can be integrated to pinpoint the molecular determinants and provide a platform to improve crop yield and quality traits in orphan crops.^80^

**Figure 3.**
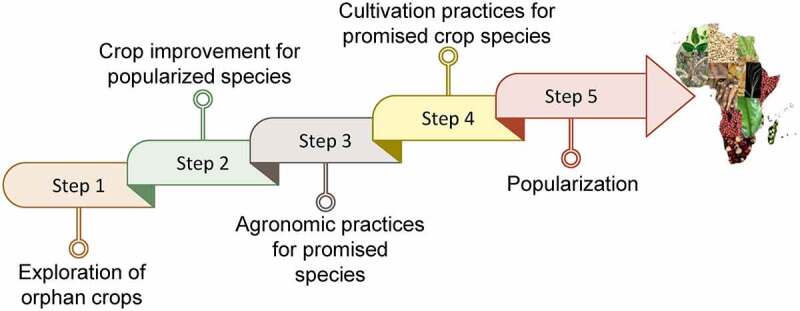
Displays the rich salient attributes of orphan crops fitting to human health and sustainable agricultural development for food and nutritional security.

The genome of orphan crops was well annotated by whole-genome sequencing projects that were often coupled with generating respective transcriptomes. The transcriptomes that were mainly preferred for annotation were taken from the same species as was done for the African eggplant,^81^ wild mustard (*Brassica juncea*),^82^ and tef (*Eragrostis tef*)^28^; however, some other cases were also seen where there was lack of resources, existing transcriptome of a close relative or well-annotated transcriptome of a model crop was used as seen in finger millet, the genome was annotated by using data from maize.^30^ Some of the other transcriptomes of these crops have also been generated in response to specific biological questions, and RNA sequencing (RNA-seq) has become the method of choice.^83^ Researchers identified at least 2416 differentially expressed genes while profiling for response against salt stress in quinoa (*Chenopodium quinoa*).^84^ Transcription analysis was done in jute-mallow to identify drought stress-related genes.^73^ Before the introduction of next-generation sequencing (NGS), microarrays were the methods of choice for transcriptome analysis. They were applied in different orphan crops to detect expression profiles relevant to abiotic stress resilience. These crops of interest include buckwheat,^85^ tef,^85^ white lupine,^86^ African nightshade (*Solanum nigrum*),^87^ and wild mustard.^88^ Epigenetics is a branch of science that deals with studying heritable gene regulation. The epigenetic changes do not involve DNA sequences but occur by the modifications caused by DNA methylation or post-translational modification of histone tails. These changes are believed to play an important role in gene expression and plant development under stress conditions (Fig. 3). The most important genomic approach, ChIP-chip (followed by microarray hybridization), was mainly used for analyzing epigenetic changes. Still, in recent years this approach was replaced by NGS technologies with ChIP-sequencing (ChIP followed by direct sequencing). Studies have shown that several epigenetic changes have contributed to crop domestication.

Several epigenetic modifications have been found to contribute to crop domestication; for example, the colorless non-ripening locus in tomatoes is induced by silencing an epiallele of a SQUAMOSA promoter binding protein-like transcription factor.^89^ Epialleles have also been linked to melon sex,^90^ rice plant height,^91^ cotton photoperiod sensitivity,^92^ and oil palm somaclonal variation.^92,93^

In addition, genome-wide analyses of the methylomes of significant crop species indicate that the bulk of these alterations is substantially conserved within a species.^94^ The use of metabolomics in the study of domestication is relatively new, but it is widely utilized in quantitative trait loci (QTL) investigations^95^; it is becoming a mainstay for understanding the genetics of quality traits in crops.^96,97^ Nonetheless, two studies address how domestication and agricultural development influence the metabolome. First, a comprehensive analysis of alterations in primary metabolism in farmed wheat and its progenitor species revealed that the domestication of emmer and durum wheat, respectively, is accompanied by changes in unsaturated fatty acid and amino acid content.^98^ Secondly, a multi-omics analysis of fruit from several hundred tomato genotypes demonstrates how domestication has affected the metabolite composition of this fruit.^99^

## 5. The Role of Genome Editing Technologies in the Orphan Crops Improvement

Genome editing has proven to be a powerful tool for crop improvement and functional genomics. Several gene-editing techniques have been widely used, including zinc-finger nucleases (ZFNs), transcription activator-like (TAL) effector nucleases (TALENs), and CRISPR/Cas9 and Cpf1. The aim of these techniques relies on the future improvement of plant sciences and the rapid remodeling of crops. Genome editing via CRISPR has become popular because of its versatility, potency, adequacy, and simplicity.^23,100,101^

Improvement and domestication of crops have become possible due to the availability of genomic information and efficient genome editing tools^102^ for the domestication of these crops. Genome editing technologies played an important role in improving orphan crops by identifying important mechanisms and gene targets for domestication and making it possible to modify these crops in a targeted manner. CRISPR-mediated genome editing has proven more advantageous as it produces potentially ‘transgene-free’ crop varieties and provides a platform to create genetically modified varieties identical to conventionally bred crop varieties. For several cultivated species, the path of domestication has become evident due to the noble intervention of genetic and genomic analyses.^57^ Editing plant genomes with extreme precision and accuracy are achieved by combining genome editing technologies, viz. CRISPR/Cas9. The CRISPR system is not entirely accepted as its acceptance is controversial, but despite this, it still holds great potential in improving crop varieties and improving farmer’s livelihood. DNA manipulation via CRISPR/Cas9 can be done in many ways like by causing random mutations (insertion or deletion) via non-homologous end joining for disruption of genes by the involvement of precise base editors to generate targeted point mutations or by whole-gene insertion employing the cell’s homology-directed repair pathway.^100^ Multiplex approaches enabled researchers to edit multiple loci simultaneously and made it possible to incorporate multiple traits at once.^103^

Domestication of orphan crops is a complicated process as it requires well-elucidated genome sequence that gives a clear understanding of paralogue structure and expression of genes, and a delivery system for genome editing. Among them, a transformation system is the simplest. Due to multigenic regulation of the same traits in orphan crops, their domestication is somehow limited.^104^ Many domestication genes like that control flowering and fruit development, increase harvest index (more product per plant), facilitate harvesting by inhibiting abscission of fruits, or make the final product easier to store, chew, and digest have been identified in many crop species.^102^ Studies have shown that mutations played an important role in altering the function of a few selected loci, known as domestication genes. Also, it has been revealed that many domestication traits possess a Mendelian inheritance pattern involving gain-of-function or loss-of-function mutations, which made it possible to modify/reconstruct these traits into suitable ones with the help of genome editing technologies, viz. CRISPR/Cas9 genome editing technology.^105^ First, the applications of CRISPR/Cas9 were limited to creating only deletion. Still, modern variants of CRISPR-based genome editing technologies are more advantageous due to new modifications and can produce targeted insertions, exchange amino acids, and modulate gene expression. The variants generated by manipulating just a small number of loci were best suited to full agricultural exploitation. Also, they demonstrated the feasibilities for crop improvement that can be achieved through a combination of genomics and gene editing. Knockout may be inadequate in many cases and may need subtler alleles like altering promoter activity or protein structure. Production of alleles with new and valuable expression properties can be achieved by editing promoter segments.^106^ Manipulating several genes with the connected combinatorial challenge of testing many variables leads to the acceleration of domestication. Hence, genome editing has been a powerful tool for domestication of wild plants and reuniting lost but desirable traits that include nutritional features or stress tolerance, yield potential, and other agronomically valuable characteristics.^107^

With the CRISPR/Cas9 approach, some successful cases of orphan crop breeding and domestication of wild relatives have been reported recently. Scientists developed genomic resources and efficient transformation methods for orphan crop groundcherry (*Physalis pruinosa*) belonging to the Solanaceae family. They then mutated the genes orthologues to tomato domestication and improved genes using the CRISPR-Cas9 system to improve productivity-related traits.^108^ The targeted genes include plant architecture, fruit size, and flower production. Studies have shown that manipulation via CRISPR/Cas9 in wild tomato (*S. pimpinellifolium*) involved editing six loci that were important for yield and productivity in present-day tomato crop lines.^109^ These modifications proved very beneficial, like increased fruit size threefold, fruit number tenfold, and a two-fold increase in fruit lycopene accumulation compared to its wild parent. CRISPR/Cas9 system gained prominence, and it has also been seen that through this approach, several types of orphan crops can be modified and have been successfully used in foxtail millet,^110^ green foxtail,^111^ and cassava.^112^

### 5.1. Prospective Applications of CRISPR System in Cassava Breeding: A Case Study

Cassava (*M. esculenta*), which is an important staple food, is grown worldwide and is a significant orphan crop not only for providing food security to tropical and subtropical regions but also being the predominant raw material for the starch industry and also provides up to 50% of the total intake calories for over 800 million people worldwide.^113^ It possesses many resilient characteristics, like being tolerant to unpredicted drought, growing very well in poor soils, and can be harvested any time of the year. Also, it is advantageous as its tubers can be retained for up to two years in soil without rotting.^114^ Despite possessing many properties, its yield is low, so many attempts are made to increase its yield, but compared to mainstream crops like rice, in cassava, there are only a few studies on the validity of the CRISPR technique. Recently many genome editing projects involving CRISPR/Cas9 have been completed to increase the yield of cassava, including disease resistance, herbicide tolerance, rapid flowering, and reduced cyanide content leaves and roots.^115^ Disease-causing pathogens majorly cause the loss of yield in cassava. Up to 50% of the total yield loss is mainly caused by the African cassava mosaic virus (ACMV) and cassava brown streak disease (CBSD). So to overcome these problems, different strategies have been developed, viz. targeted mutation using Cas9/gRNA.^116^ Gene editing of two isoforms of elF4E, *nCBP-1* and *nCBP-2*, were done simultaneously, resulting in heritable delayed and suppressed CBSD aerial symptoms and reduced severity and frequency of storage root necrosis. Resistance against ACMV in cassava was achieved by knockout of the AC2 gene resulting in 33–48% evolution of the gene by forming transgenic lines. Studies have also shown that during glasshouse inoculation CRISPR system does not provide effective virus resistance.^112^

CRISPR/Cas9-mediated gene insertion and replacement techniques have majorly developed herbicide-resistant crop varieties. In *cassava*, herbicide resistance was developed by HR and NHEJ DNA repair pathways by substituting crucial amino acids in the conserved domain of 5-enolpyruvylshikimate-3-phosphate synthase in order to give resistance against glyphosate-based herbicides (EPSPS)^117^ and produce phenotypically normal glyphosate tolerant cassava. This method also provided knowledge about gene editing techniques for the further modification and improvement in cassava.^118^ Recent studies have also shown that many attempts were made to develop cassava that can be used to visualize the early stages of CBB infection in vivo.^119^ CRISPR-mediated homology-directed repair (HDR) was also used to generate plants with scarless insertion of GFP at the 3’ end of the CBB susceptibility (S) gene *MeSWEET10a*. These are successfully visualized at transcriptional and translational stages.

### 5.2. Trait Improvement via CRISPR/Cas9

In cassava, two isoforms of *MESSIII* genes, *MESSIII-1* and *MESSIII-2*, were mutated simultaneously using CRISPR/Cas9 system, resulting in cassava with edited genes related to the starch synthesis pathway.^110^ This research provides a platform to examine the role of genes in regulating amylopectin glucan synthesis in cassava. Studies have also shown that two amylose synthesis genes, PTST1 and GBSS, can reduce or remove amylose content in root starch by using CRISPR/Cas9-mediated targeted mutagenesis. Flowering in cassava was also accelerated by incorporating the *Arabidopsis* FLOWERING LOCUS T gene into the genome editing cassette, which was generally unusual in glasshouse conditions.^120^ Researchers also achieved activated acceleration of cassava flowering in cassava by using CRISPR/Cas9 mediated disruption of Multiple TFL-like Floral Repressors.^121^

During cotyledon-stage somatic embryogenesis, the mutants were phenotypically albino, which provided a good idea about gene’s role in plants using CRISPR/Cas9-mediated genome editing technology to target the phytoene desaturase (MePDS) gene in cassava. Using CRISPR/Cas9-mediated genome editing technology to target the phytoene desaturase (MePDS) gene in cassava, researchers could determine the relevance of the gene in the plant due to the albino phenotype of mutants during cotyledon-stage somatic embryogenesis. This prevented the requirement for gene sequencing to prove that a mutation had happened in the target gene.^122^ Therefore, it provided a helpful arena for testing and enhancing CRISPR/Cas9 and other genome editing methods in cassava.

## 6. Different Strategies to Domesticate Orphan Crops to Develop Future-ready Crops

**Figure 4.**
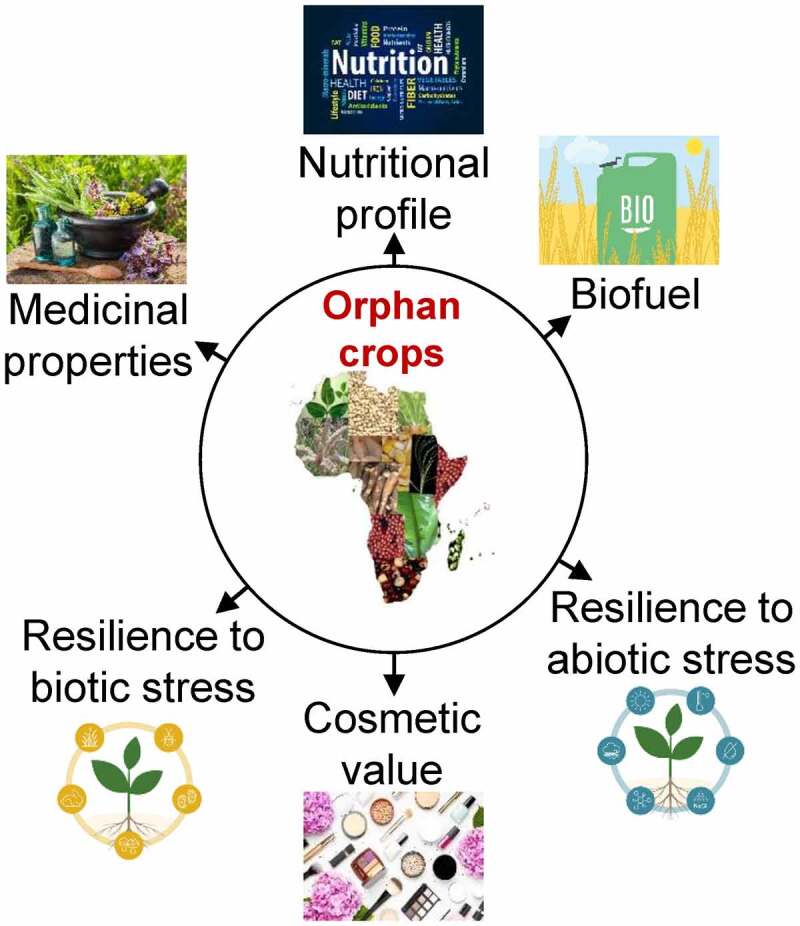
Showing the stepwise strategies to popularize the orphan crops for food and nutritional security.

Most of the world’s population depends on a few species of crops like rice, maize, wheat, potato, and soy to meet the food and nutritional demands, so research and breeding efforts are mainly focused on these crop varieties. Some varieties of tasty, nutritious, and well-adapted orphan crops are also grown. Still, their cultivation is limited cause of their wild characteristics and can be domesticated by following strategic steps (Fig. 4). Many attempts were made to explore the domestication of these orphan crops, most suitable for genome editing techniques. Genes of *P. pruinosa* (groundcherry), an orphan crop, are modified to explore its domestication, especially the modification of those genes carried out whose orthologues control the domestication traits in the close relatives.^108^ The results showed the power of this approach and demonstrated the importance of identifying mechanisms and gene targets. The path of domestication from wild ancestors to modern crops has become possible for several cultivated species with the help of genetic and genomic analysis.^102^ Studies have shown that mutations that alter the functions of a few selected loci, known as domestication genes, have played a determining role. Wild teosinte and modern maize alleles at a few major loci are responsible for much of the difference.^102^ Several domestication genes control flowering and fruit development, inhibiting the abscission of fruits and thus facilitating harvesting, increasing harvest index (more product per plant), and making the final product easier to store, chew, and digest have been found in different crop species. Manipulating these traits has proven an outstanding achievement in revolutionizing the domestication of different crop species.^102^ Neolithic gatherers selected other traits like loss-of-shattering unintentionally. Increased food availability and domestication of crops enabled the flourishing of sciences, arts, and technology. Flourishing of sciences, arts and technology have emerged by the increase in food availability and domestication of crops. Most of the improvements of our stable crops are based on Neolithic selection, but recently other improvements are also seen, like semi-dwarfism in wheat and rice. The combination of spontaneous mutation in SELF-PRUNING in tomato was radically altered to enable its mechanical harvesting.

The availability of genomic information and efficient genome editing tools played an important role in exploring crop domestication and improvement.^18,102^ Through these modifications, wild species and unimproved crops can also be modified in a targeted manner to produce novel and improved crops. An orphan crop groundcherry (*P. pruinosa*), which belongs to the solanaceous species, produces small but tasty berries. Due to its wild characteristics like sprawling habit, husked and small fruit, and strong fruit abscission, it cannot be grown on an agricultural scale.^123^ Researchers saw an opportunity: is it possible to achieve the corresponding gains in this sister species by modifying the known gene targets of tomato domestication. They increased the number of flowers and delimited flowering time on both primary and axillary shoots by targeting repressors of the florigen pathway by gene editing techniques.^108^ Knocking out many genes has shown great results, like knocking out a classical improvement gene; SELF-PRUNING, which controls indeterminate versus determinate growth in tomato, has proven very beneficial and resulted in extreme compactness. Knockout of another gene, SP5G, a florigen repressor, has increased axillary flowering and fruit destiny, although it does not affect a primary shoot.^108^ Scientists also targeted the pathway which regulates shoot apical meristem size by the interaction of CLV3, a small peptide with its receptors (CLV1 and others) known as the CLAVATA pathway. Knocking out of CLV1 had shown effective results, viz. flower meristem size was increased, additional flower organs and two-locule fruit was converted to larger, three-locule fruit. These modifications generate variants most suited to full agricultural exploitations. These manipulations also gave a great idea about the possibilities of combining genomics and gene editing of just a small number of loci.^108^ These studies also demonstrate the challenges that ‘domesticators will encounter.

Most important is to predict those targeted modifications that will generate the ideal phenotype. By understanding the domestication history of crops closely related to orphan crops, information about the target identity can be achieved. The structure of gene networks varies per node number, type, and connection.^124^ Breeders and geneticists have identified that genetic modifiers present in the population can dramatically alter mutation phenotype. It was demonstrated by dwarfing effect in groundcherry by SELF PRUNING knockout or by the inability of SP5G manipulation to modify primary shoot flowering. Knockout may not be sufficient in some cases and may need subtler alleles like altering promoter activity or protein structure. Production of new alleles with new and valuable expression properties can be achieved by editing promoter segments.^106^

Due to multigenic regulation of the same traits in orphan crops, their domestication is limited.^104^ All things were considered that were important for taming of wild species. The work carried out by Lemmon et al. explained the approach’s feasibility and the importance of investing in research and enhanced information on genomes, genes, and cellular mechanisms behind plant traits. Some orphan crops serve as better candidates for gene manipulations than others, and some traits will be easier targets than others. This information and essential tools for manipulating DNA may serve as the ingredients for success. Accelerated domestication involves the manipulation of several genes with the connected combinatorial challenge of testing many variables.

Lastly, the faster domestication envisioned here may include the manipulation of several genes and the associated combinatorial difficulty of evaluating numerous factors. In reality, domesticated species may owe at least some of their success to their greater ease of genetic manipulation. If crucial domestication features were monogenic and variable in the progenitor, they would have been readily evident and selected by breeders. Optimistically, faster domestication will be an essential component of the survival toolkit, the collection of technologies required to sustain human civilization. At a minimum, understanding how domestication genes function in various animals will improve established crops.^104^

## 7. Available Genetic Improvement Methods for New and Orphan Crops

### 7.1. Advanced and Conventional Breeding

The suitable approaches for genetic improvement and modification of any particularly new or orphan crop depend on the ideotype targets, good knowledge about inheritance, and genetic architecture of defining traits. The marker-assisted selection was widely used to develop major crops and has also begun to be applied to minor crops, that is, orphan crops. Examples of annual orphan crops include pigeon pea^26^ and foxtail millet,^111^ as also a wide range of perennial plants.^125,126^ However, in most cases, relatively high costs of phenotyping remain a constraint.^59^

In most instances, the comparatively high costs of phenotyping continue to be a barrier.^59^ This is especially true for perennial crops, whose evaluation requires several years of growth and whose huge life forms necessitate extensive area in field trials. Using genome-wide association scans, Cichy et al.^59^ revealed genomic sites linked with variance in the so-called ‘cooking time characteristic’ in a common bean diversity panel.^127^ Due to relatively substantial research expenditures, the common bean is arguably not precisely an orphan crop, but it is emblematic of other orphan legumes. The discovery of connections between some genomic regions and cooking time in legumes is significant since extended cooking durations restrict the seed’s efficiency as a food source.

Cichy et al.^59^ found statistically significant associations between cooking time and single nucleotide polymorphisms (SNPs) on three *Phaseolus vulgaris* chromosomes, with the greatest connections seen on chromosome 6 (Pv06). Pv06 had two homologous cation/H^+^ exchanger genes, one homologous to *AtCHX3* and the other to *AtCHX4*. On cassava (Manihot esculenta), a vegetatively propagated orphan annual root crop, the efficiency of genomic selection is now being studied.^128^  

Finger millet (*Eleusine coracana*), a seed-propagated annual grain, is the second example of an orphan crop for which this method is being investigated. As with conventional marker-assisted selection, the lack of phenotypic data from suitable training populations is the most significant barrier to applying genomic selection to novel and orphan crops.^59^ However, implementing this strategy might be particularly beneficial for slow-maturing perennial novel and orphan crops that are difficult to phenotype directly for crucial production features.^125^ When the underlying biological basis of crucial features is poorly understood, as with many novel and orphan crops, genomic selection may be very successful.

### 7.2. Speed Breeding

Another approach now being applied to orphan crops is speed breeding, which reduces the generation interval in breeding programs by modifying the photoperiod exposure of daylength-sensitive plants to accelerate their development (typically by prolonging ‘long-day’ plants’ exposure to light.^129^ Each year, the number of probable generations has been extended from three to six for the long-day annual legume chickpea (*Cicer arietinum*).^130^ Annual grain amaranth crops with “short days” have also proven successful.^131^ Speed breeding should be especially beneficial when used with genomic selection, since this enables for selection during rapid cycling when complete phenotypic data is unavailable.^132^ Transportable ‘speed breeding capsules,’ consisting of shipping containers retrofitted with temperature and light controls, irrigation systems, and greenhouse benches, have been proposed to lower the expenses of the speed breeding strategy for novel and orphan crops in low-income countries.^9^

### 7.3. Participatory Breeding and Selection

In high-income countries, ‘citizen science’ programs that analyze crop germplasm have proven effective, as demonstrated by,^133^ who investigated genotype-environment interactions in soybean (*Glycine max*) using data gathered by 1800 gardeners across Germany. Low-income countries, with larger rural populations and many engaged small-scale farmers, have an even greater opportunity for participatory experimentation. These communities may meaningfully analyze genetic materials in various target habitats and cropping systems and give additional information on crop yield and consumption if they are appropriately maintained. Participatory domestication approaches, for example, have been effectively used to genetically develop novel and orphan fruit tree crops in Central Africa, such as the semi-domesticated safou (*Dacryodes edulis*) and the incipiently domesticated bush mango (Irvingia gabonensis and I. wombolu).^133^ The participative technique is particularly beneficial in low-income countries, where production circumstances are variable, and crop preferences are little recognized.^134^ Both of these circumstances frequently apply to new and orphan crops.

Environmental considerations ‘Landscape genomic’ methods for crop growth are particularly important for perennial plants, which are largely wild populations that have evolved to local abiotic circumstances over many generations.^135^ This is because the ‘in situ’ decision-making process saves time and effort compared to traditional field testing. Genomic data from plants growing in wild populations is connected with environmental factors using statistical approaches that account for the underlying adaptively neutral genetic structure induced by genetic drift.^136^ Established correlations may be theoretically utilized to screen larger germplasm panels for favorable allele compositions for specific production circumstances. The enormous amount of georeferenced interpolated environmental data sets currently available digitally, such as temperature and precipitation profiles^137^ and soil types, make comparisons easier^138,139^; for example, using soil maps to identify the soil environment as a critical driver of adaption in a landscape genomic analysis covering the native range of barrel medic (*Medicago truncatula*, a legume), with a high number of SNPs associated with soil variables, including SNPs in candidate genes involved in nodulation/symbiotic nitrogen fixation. The landscape method may be used for orphan crop landraces and new and orphan crops’ wild germplasm if local adaptation is presumed to have happened throughout orphan crop development and ecogeographical range expansion. A meta-analysis of various crop progenitors and landraces in the same geographical location might give comparative insights into natural and human adaptation mechanisms in this scenario. Statistical methods that link the results of many common garden genome-wide association studies, which look into the genetic basis of phenotype–trial site associations, with wild and/or landrace sample environment–genomic correlations are now available.^140^ This will aid in the knowledge of causative loci for adaptability and the development of appropriate tactics for new and orphan crop range extension.

## 8. Engineering Insect-Pest Resistance through Exploiting Crop Wild Relatives

Studies on crop wild relatives (CWRs) have explained the loss of resilience traits that had occurred during the process of crop domestication (manmade selection) and is also known as “domestication syndrome,” according to which human needs were fulfilled by the developing crops which in turn made these crops susceptible and were not able to combat environmental fluctuations. According to these studies, most germplasm-cultivated lack the ability to manage insect attacks.^141^ However, breeding for insect resistance using CWRs may prove a feasible strategy to increase the genetic diversity of the primary gene pool. Still, it has limitations as it remained unsuccessful in most of the crops due to the following reasons such as biological barriers, cross-incompatibility, linkage drag, and sterile embryo production. Different approaches like advanced genetic engineering tools such as transgenesis and genome editing were mainly used to introduce novel traits from CWRs into cultivated species. One of the essential aspects that are responsible for insect resistance is the identification of genetic variation. Comparative transcriptome and proteome analysis in response to insect feeding has proven the effective strategy for the identification of sequence and expression level polymorphism, where there is no feasibility of introgression. A tangible approach to introducing variability in the cultivated crops can be made by editing their genes which is based on the respective variation in CWRs, and it can be made feasible by using multi-omic strategies, firstly evaluating the variation in the sequences of relevant insect-responsive genes between the susceptible cultivated germplasm and the resistant wild relative. These can be successfully utilized for genome editing after validating resistance genes against the relevant pests. These approaches have successfully provided resistance in the cultivated gene pool to combat insect pests. Increasing research activities provided a platform for genome editing for insect pest management.^141,142^, Resistant phenotypes using sequence variation in economically important crops can be developed using overexpression or silencing strategies.^142^ However, developing resistance via genome-editing-based sequence variation has not yet been proved.

Crop wild relatives include the progenitors of crops and other species that are more or less closely related to them. They provided plant breeders with a broad pool of potentially valuable genetic resources, thus proving beneficial to modern agriculture. Significant advances have been made after the 20 years of the Prescott-Allens’ study, which was based on molecular technologies and hybridization procedures available for breeding and cultivar development, that allowed the incorporation of more distantly-related taxa, and in our knowledge of the wild relatives available for use in these programs. The beneficial traits conferred by CWR genes included over 80% associated with pest and disease resistance. For over a century, breeders used wild relatives to get resistance to diseases^143^ and also searched extended gene pools for the genes that would provide resistance to major crop pests and diseases.^144,145^, They incorporated wild genes into about 13 crops, and all except barley and chickpea became disease resistant after the incorporation of wild genes. Since then, the use of this approach has increased steadily. Wild resistant traits were also used in tomatoes to develop disease resistance. It has been reported that this approach has been used at a rate of about one per year since 1982.^138^ It has also been proven that the disease-resistant genes currently used in commercial cultivars have been bred from wild genetic resources (D. Zamir, personal communication). Fungicides have limited effectiveness against the pathogen, so growing lettuce in many parts of Europe may not be possible without introducing genes. However, resistance genes overcome these problems rapidly, and the breeders constantly return to wild germplasm for new resistance genes.^146,147^

## 9. Success Achieved in the Breeding of Orphan Crops

Since the beginning of the twenty-first century, unprecedented changes have occurred in plant breeding techniques. Some techniques that have contributed to the modernization of some of the crops in some regions include genotyping and phenotyping technologies, genomics, and analytics. Major crops have significantly contributed to the needed global increase in agricultural production, while orphan crops are global and of localized importance. The reality of orphan crops was that their research was limited. Also, there was very little knowledge available for orphan crops in genetics and genomics. Still, day by day, technologies are becoming more affordable and thus decreasing the cost of knowledge generation, often evolving the reality. Several international initiatives that were available for breeders, like the Generation Challenge Programme (GCP, www.generationcp.org/sunsetblog),^27^ provided breeders access to genetic and genomic technologies for some orphan crops and also the knowledge previously available only for large commercial crops.^26^ Different achievements seen in different crop species include characterization of genetic diversity,^148^ understanding the basis of the genetics of agronomic traits,^149^ and identification of elite alleles at target genes^150^ for introgression in elite germplasm to impact crop performance.^151^ Advancements in sequencing orphan-crop genomes have been significantly achieved over the last decade^61^ and also focus on developing tools aimed at discovering and characterizing loci and genes of use in molecular breeding of those sequenced species.^152^

## 10. Digitalizing Breeding and Providing Support

Many attempts were made to modernize breeding techniques, and the modernizing approaches without a reliable data management system are very risky. Several research projects and breeding programs failed to achieve their goals due to poor data quality, lack of documentation, or lost institutional memory. Digitalization of breeding has brought several improvements, such as increased effectiveness of seed management, data capture, quality control, documentation, and analysis. More accuracy can be achieved in the selection decisions at all stages of the breeding process.^152^ It also contributed to establishing routines to standardize the storage of germplasm information that include pedigree, phenotypes and genotypes, breeding protocols, metadata (location, climate, etc.), and trait oncology, which in turn provide different benefits such as enabling data mining and also sharing of opportunities across a broader range of environments and teams.^153^

## 11. Conclusions and Future Outlook

Recalling orphan crops, negligible and underutilized crops that are not frequently operated globally but often play greater agricultural roles more in the area. These crops would deliver not only a more varied food organization less susceptible to to climate-induced inadequacies but also nourishing and stable food alternatives for the future. Genome editing of orphan crops could explore the domestication of these species. Domesticating an orphan crop plant requires multiple tools: a well-elucidated genome sequence, including the understanding of paralogue structure and gene expression, and a delivery system for genome editing, the simplest being a transformation system. Orphan crops have recently gained prominence for being stress tolerant and nutritious. Apart from being nutritious, these orphan crops can be grown with little agricultural input. Also, being stress-resilient, these crops are safe from the various vagaries of climate change and ensure yield even when other major crop varieties fail. Genome editing holds great promise for increasing crop productivity. There is particular interest in advancing breeding in orphan crops, which are often burdened by undesirable characteristics resembling wild relatives – developing genomic resources and efficient transformation methods in the orphan crops and using CRISPR/Cas9 to mutate orthologues of crop domestication and improve genes that control plant architecture, flower production, and fruit size, thereby improving these major productivity traits. Thus, translating knowledge from model crops enables the rapid creation of targeted allelic diversity and novel breeding germplasm in distantly related orphan crops. Genome editing could be used as a novel platform by plant breeders to speed up the domestication of semi-domesticated or even wild plants, creating a more diverse base for the sustainable provision of food and fodder in the future. Further domestication of underutilized crops may pave the way for rapid global food security and higher crop yields.
